# Tracking of maternal self-efficacy for limiting young children’s television viewing and associations with children’s television viewing time: a longitudinal analysis over 15-months

**DOI:** 10.1186/s12889-015-1858-3

**Published:** 2015-05-30

**Authors:** Jill A Hnatiuk, Jo Salmon, Karen J. Campbell, Nicola D. Ridgers, Kylie D. Hesketh

**Affiliations:** grid.1021.20000000105267079Centre for Physical Activity and Nutrition Research, Deakin University, 221 Burwood Highway, Burwood, VIC 3125 Australia

**Keywords:** Television, Infant, Toddler, Maternal behaviour, Tracking

## Abstract

**Background:**

Mothers’ self-efficacy for limiting their children’s television viewing is an important correlate of this behaviour in young children. However, no studies have examined how maternal self-efficacy changes over time, which is potentially important during periods of rapid child development. This study examined tracking of maternal self-efficacy for limiting young children’s television viewing over 15-months and associations with children’s television viewing time.

**Methods:**

In 2008 and 2010, mothers (n = 404) from the Melbourne InFANT Program self-reported their self-efficacy for limiting their child’s television viewing at 4- and 19-months of age. Tertiles of self-efficacy were created at each time and categorised into: persistently high, persistently low, increasing or decreasing self-efficacy. Weighted kappa and multinomial logistic regression examined tracking and demographic and behavioural predictors of change in self-efficacy. A linear regression model examined associations between tracking categories and children’s television viewing time.

**Results:**

Tracking of maternal self-efficacy for limiting children’s television viewing was low (kappa = 0.23, p < 0.001). Mothers who had persistently high or increasing self-efficacy had children with lower television viewing time at 19-months (β = −35.5; 95 % CI = −54.4,-16.6 and β = 37.0; 95 % CI = −54.4,-19.7, respectively). Mothers of children with difficult temperaments were less likely to have persistently high self-efficacy. Mothers who met adult physical activity guidelines had 2.5 greater odds of increasing self-efficacy.

**Conclusions:**

Interventions to increase and maintain maternal self-efficacy for limiting children’s television viewing time may result in lower rates of this behaviour amongst toddlers. Maternal and child characteristics may need to be considered when tailoring interventions.

## Background

Limiting the time that children spend watching television is recommended for minimizing negative health outcomes such as increased adiposity and poorer psychosocial health and cognitive development [1, 2]. As such, current guidelines recommend that children under two years of age do not engage in any television viewing [3, 4]. However, by 3 months of age, 40 % of American children watch television, and this proportion increases to approximately 90 % by 24 months of age [5]. As mothers play an important gatekeeper role in their child’s health behaviours [6], understanding how mothers perceive their ability to facilitate or limit these behaviours and how this changes over time is likely to provide valuable insights into the development of effective interventions to promote children’s health behaviours.

Mothers’ self-efficacy to support or limit her children’s health behaviours is likely to be an important influence [7–9]. Self-efficacy refers to an individual’s confidence to engage in a particular behaviour under certain conditions [10]; for example, a mothers’ confidence in her ability to limit her child’s television viewing time when the child is fussing. Cross-sectional evidence suggests that maternal self-efficacy for limiting children’s television viewing is associated with less television viewing time [7, 8] as well as a lower likelihood of exceeding screen time recommendations [11]. However there is also evidence that mothers’ self-efficacy may decrease over time [8, 11]. Mothers of younger children have reported higher self-efficacy [8] and higher optimism [12] about their ability to influence their child’s television viewing behaviours than mothers of older children suggesting that as children get older this may result in decreased maternal self-efficacy to limit their television viewing [8]. However, to date no longitudinal research has confirmed this.

The concept of tracking refers to an individual’s rank order position relative to others in the same cohort over a period of time [13]. In this context, it would signify how mothers’ self-efficacy is maintained in rank order relative to her peers. If high tracking exists, this can be useful for predicting which mothers may need to be provided with greater support from early in their child’s life as this low self-efficacy will tend to persist as their child grows up. However, should maternal self-efficacy change over time, an examination into patterns of movement (e.g. increasing or decreasing self-efficacy) may be necessary.

By identifying characteristics of mothers who are at risk of having low self-efficacy or decreasing self-efficacy over time it may be possible to identify mothers who would benefit from having greater support and strategies available to increase and/or maintain their self-efficacy. Maternal psychological states (e.g. anxiety, depression, education level) and child characteristics (e.g. temperament) are known to influence maternal self-efficacy generally [14, 15], yet few studies have examined self-efficacy in relation to children’s television viewing specifically (domain-specific self-efficacy). The only variable identified that has been associated with maternal self-efficacy for limiting television viewing is maternal self-reported physical activity level [16]. However, previous research has found that both maternal and child demographic characteristics (e.g. lower maternal education, older child age) and maternal and child behaviours (e.g. higher maternal television viewing time and negative child temperament) are positively associated with children’s television viewing time [17–19]. It is therefore possible that these factors also explain changes in mothers’ self-efficacy for limiting television viewing behaviours across the early childhood period.

Therefore, the purpose of this study was to examine the tracking of maternal self-efficacy for limiting young children’s television viewing and associations with children’s television viewing time. A secondary aim was to identify demographic (mothers’ education level, child age) and behavioural (mothers’ television viewing time and physical activity level and child temperament) predictors of mothers who maintained, increased or decreased their self-efficacy for liming television viewing over the first two years of their children’s lives.

## Methods

### Participants and procedures

The participants for this study were drawn from the Melbourne InFANT Program, a cluster-randomized controlled trial promoting obesity-protective behaviours in young children. The trial was delivered to 542 first-time mothers from when their children were 4-months of age and ended when their child was 19-months of age. No intervention effects were observed for maternal self-efficacy for limiting television viewing, therefore participants from both the intervention and control groups were included for the purpose of this study. Self-report questionnaires were completed by mothers at baseline in 2008 (T1- child aged 4-months) and program conclusion in 2010 (T2- child aged 19-months). The study was approved by the Deakin University Human Research Ethics Committee and the Victorian Government’s Office for Children. All participants provided informed consent prior to taking part in the study.

### Measures

#### Demographic and behavioural predictors at T1

Mothers reported their highest level of education (categorized as ‘low’ [secondary school or lower], ‘medium’ [trade/certificate qualification], or ‘high’ [university degree or higher]), their age, and the age and sex of their child.

Child temperament was reported by mothers using a single item from the Australian Temperament Project [20]. Mothers rated on a 5-point Likert-type scale the difficulty of their child relative to other children (much easier than average = 0; much more difficult than average = 4). As few mothers reported their child was ‘much more difficult than average’, this response category was combined with the ‘more difficult than average’ response category for use in the analyses.

Mothers’ physical activity was assessed using the Active Australia Survey, a reliable and valid measure of self-reported leisure-time physical activity [21, 22]. Following instrument protocols [21], total physical activity (mins/week) was determined by summing the time spent walking (>10 minutes), the time spent in moderate-intensity physical activity (MPA) and twice the time spent in vigorous-intensity physical activity (VPA) in the past week. Minutes spent in any given activity intensity were truncated at 840 minutes/week and time spent in MPA and VPA combined (MVPA) was truncated at 1680 minutes/week [21]. Based on the average time spent in MVPA per week mothers were either categorized as meeting (≥150 mins/week) or not meeting (<150 mins/week) physical activity recommendations.

Maternal television viewing time (mins/week) was assessed for weekdays and weekend days by the questions, “On a usual weekday (weekend day), about how many hours do you usually spend sitting down and watching television or videos/DVDs?”, previously shown to be valid and reliable [23]. Television viewing time (mins/day) was calculated using a weighted average between the weekday and weekend day responses. Total television viewing times reported were truncated at 1060 minutes/day. In absence of television viewing guidelines for adults, maternal television viewing time was retained as a continuous variable.

#### Maternal self-efficacy for limiting children’s television viewing at T1 and T2

Maternal self-efficacy for limiting children’s television viewing was assessed using three items from a previously developed scale as no measures to assess self-efficacy in these specific domains were published at the time of assessment [8]. The questions assessed mothers’ confidence for saying ‘no’ to television/DVDs when their child was fussing, providing active play options over television viewing and keeping their baby entertained without using television/DVDs. These items had good internal reliability in this sample (α = 0.72 at T1 and α = 0.83 at T2) and acceptable test-retest reliability in a separate sample at child aged 4 months (0.56 – 0.77) and child aged 19 months (0.59 – 0.90) old. All questions examining maternal self-efficacy were scored on a 4-point Likert-type scale where 0 = not at all confident and 4 = extremely confident. Scores were then generated by taking the average score of the three items.

Data for maternal self-efficacy for limiting children’s television viewing was split into tertiles (high, mid, low maternal self-efficacy) at T1 and T2 separately. To assess the direction of change, a categorical variable was created using a similar approach to other tracking studies [24, 25]. This variable consisted of four categories: (1) persistently high self-efficacy (top tertile at T1 and T2 or middle tertile at T1 and T2), (2) persistently low self-efficacy (bottom tertile at T1 and T2), (3) increasing self-efficacy (bottom tertile at T1 to middle/ top tertile at T2 or middle tertile at T1 to top tertile at T2) or (4) decreasing self-efficacy (top tertile at T1 to middle or bottom tertile at T2 or middle tertile at T1 to bottom tertile at T2).

#### Children’s television viewing time

At T1 and T2, mothers proxy-reported the total time (hours and minutes) that their child spent watching or in front of the television during the past week. Test-retest reliability of this single item measure from in a separate sample was good (ICC = 0.84). All responses were converted to minutes/week.

### Statistical analysis

Despite no statistical differences in maternal self-efficacy for limiting children’s television viewing or tracking categories between intervention and control groups at T1 or T2, all data were adjusted for intervention group due to exposure to the program. Tracking of maternal self-efficacy for limiting television viewing was assessed using weighted kappa and multinomial logistic regression (reference category: middle tertile at T1 and T2). Linear regression analyses examined associations between maternal self-efficacy tracking categories (persistently high self-efficacy, persistently low self-efficacy, increasing self-efficacy, decreasing self-efficacy) and children’s television viewing time at 19-months old, adjusted for intervention group, baseline television viewing time and clustering by first-time mothers group. The odds of being in the different categories of movement based on maternal and child demographic and behavioural predictors were analysed using multinomial logistic regression (reference category: persistently low self-efficacy), controlling for the same covariates described above. All analyses were conducted in Stata 12.0.

## Results

A total of 404 mothers had complete questionnaire data for T1 and T2. Mothers with complete data had children who were slightly younger at T1 (on average approximately 2 weeks) than those who did not have complete data (p < 0.05). No other differences in demographic or behavioural predictors assessed in this study were observed. Demographic characteristics of included participants are outlined in Table 1.

**Table 1:** Baseline (T1) demographic and behavioural characteristics of participants (n = 404)

Child characteristics	T1
Male (%)	53.6 %
Mean (SD) age (months)	3.8 (1.3)
Child temperament (%)	
Much easier than average	15.6 %
Easier than average	40.9 %
Average	35.7 %
More/much more difficult than average	7.7 %
Any television viewing time	58.4 %
Mother characteristics	
Mean age (years)	32.3 (4.3)
Maternal education (%)	
Low (≤ secondary school)	19.6 %
Medium (trade or certificate qualification)	24.6 %
High (university degree +)	55.8 %
Physical activity (≥150 mins/week)	83.9 %
Mean (SD) television viewing (mins/week)	213.6 (141.1)

**Fig. 1:**
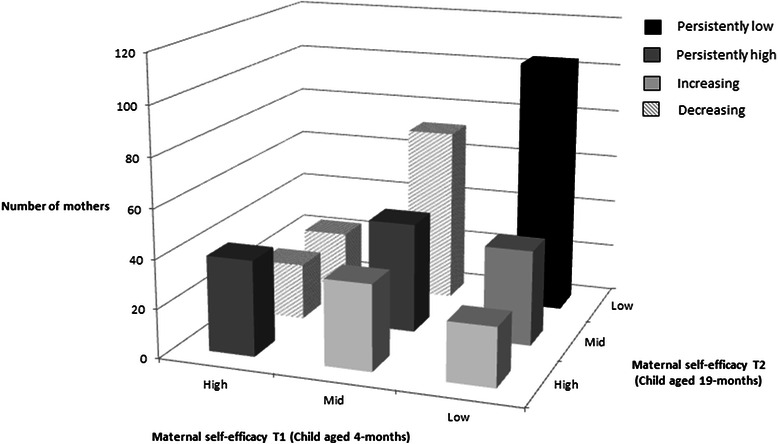
Categories of maternal self-efficacy for limiting children’s television viewing from child aged 4-months (T1) to 19-months (T2)

Although significant, the weighted kappa coefficient indicated that tracking of maternal self-efficacy for limiting television viewing was low over the 15-month time period (Κ = 0.23, p < 0.001). Compared with mothers who were in the middle tertile at T1 and T2, mothers in the top tertile at T1 were 2.2 (95 % CI = 1.03–4.61) times more likely to remain in the top tertile at follow-up. Individuals in the bottom tertile at T1 did not have an increased likelihood of remaining in the bottom tertile at T2 compared to those in the middle tertile, although the data indicated a trend in this direction (OR = 1.67; 95 % CI = 0.99–2.80). Figure 1 depicts the number of participants within each category of maternal self-efficacy at each time point. The greatest proportion of mothers (~30 %) had reduced self-efficacy over time, with the lowest proportion of mothers in the persistently high self-efficacy category (~20 %).

Table 2 reports the associations between the maternal self-efficacy tracking categories and children’s television viewing time at 19-months old. Compared to those mothers with persistently low self-efficacy, mothers who had persistently high self-efficacy or increasing self-efficacy had children with lower television viewing behaviour at 19-months (β = −35.5 mins; 95 % CI = [−54.4,-16.6] and β = −37.0; 95 % CI = [−54.4,-19.7], respectively).

**Table 2:** Associations between maternal self-efficacy tracking categories and children’s television viewing time (mins/week) at 19-months old

Maternal stability	β (95 % CI)
Persistently low self-efficacy	Ref.
Persistently high self-efficacy	−35.5 (−54.4, −16.6)
Increasing self-efficacy	−37. 0 (−54.4, −19.7)
Decreasing self-efficacy	−2.6 (−22.9, 17.7)

Linear regression analyses, adjusted for intervention group, baseline television viewing time and clustering by first-time mothers group

Relative to mothers who rated their child’s temperament as much easier than average, those who rated their child’s temperament as ‘average’ or ‘more difficult than average’ had 71 % and 93 % lower odds respectively of having persistently high self-efficacy for limiting television viewing compared to mothers with persistently low self-efficacy for limiting television viewing (Table 3). Additionally, mothers who met the adult physical activity recommendations had 2.5 times greater odds of increasing their self-efficacy for limiting their child’s television viewing over time compared to mothers who did not meet physical activity recommendations.

**Table 3:** Odds ratios and 95 % confidence intervals (CI)^a^ of changing maternal self-efficacy to limit their child’s television viewing according to maternal and child predictors among children aged 4- and 19-months^b^

Predictor variable	Increasing self-efficacy		Decreasing self-efficacy		Persistently high self-efficacy	
	OR	95 % CI	OR	95 % CI	OR	95 % CI
Maternal physical activity						
<150 mins/week	1.0 (ref)		1.0 (ref)		1.0 (ref)	
≥150 mins/week	2.49*	**(1.08 – 5.75)**	2.18	(0.89 – 5.32)	1.87	(0.90 – 3.88)
Maternal TV time	1.00	(0.99 – 1.01)	1.00	(0.99 – 1.00)	1.00	(0.99 – 1.01)
Maternal education						
Low	1.0 (ref)		1.0 (ref)		1.0 (ref)	
Mid	0.77	(0.34 – 1.76)	1.05	(0.50 – 2.19)	1.45	(0.57 – 3.68)
High	0.88	(0.36 – 2.15)	0.99	(0.50 – 1.99)	2.17	(0.90 – 5.27)
Child age	0.92	(0.71 – 1.18)	1.10	(0.87 – 1.41)	0.86	(0.664 – 1.15)
Child temperament						
Much easier than average	1.0 (ref)		1.0 (ref)		1.0 (ref)	
Easier than average	0.89	(0.34 – 2.30)	1.01	(0.47 – 2.15)	0.41	(0.15 – 1.10)
Average	0.43	(0.16 – 1.14)	0.58	(0.24 – 1.40)	0.29*	**(0.11 – 0.76)**
More/much more difficult than average	0.38	(0.10 – 1.47)	0.40	(0.12 – 1.37)	0.07*	**(0.01 – 0.46)**

^a^Multinomial logistic regression, adjusted for intervention group and clustering by first-time mothers group; Reference group: persistently low self-efficacy

^b^Bold results are significant at p < 0.05

## Discussion

This study was the first to examine tracking of first-time mothers’ self-efficacy for limiting television viewing in young children and associations with children’s television viewing time. This is important for our understanding of how and when interventions might be best delivered to young families to minimize children’s television viewing time. It was found that, although statistically significant, tracking of this construct was low over the 15-month period, with around half of participants moving tertiles between T1 and T2, and with a greater proportion of change occurring in a downward direction. This is consistent with cross-sectional work in the field which has found that self-efficacy for limiting television viewing is lower amongst parents of preschool children compared to parents of infants [8]. It is possible that greater movement would be evident if tracking of maternal self-efficacy was assessed over a longer period of time.

The level of tracking observed in this study varied by tertile rank, with mothers in the extreme tertiles (high or low) at baseline more likely to remain in those tertiles 15-months later. While this is a positive finding for those mothers with high self-efficacy, it is less desirable for mothers with low self-efficacy. Mothers who do not possess particularly high or low self-efficacy at baseline may be more susceptible to changes in self-efficacy, albeit in a positive or negative direction. Thus, an opportunity exists to provide support to these mothers so that the change they may experience is in a favourable direction.

Building from cross-sectional work in the field [7, 8, 11], this study found that favourable changes in maternal self-efficacy over the first year and a half of children’s lives are associated with lesser television viewing time, even after controlling for baseline television viewing time. This suggests that supporting mothers to maintain high self-efficacy or intervening to increase maternal self-efficacy over the early childhood period is a worthwhile endeavour to reduce children’s television viewing time. In particular, more intensive or targeted support strategies may need to be delivered to mothers who have low self-efficacy for limiting their infant’s television viewing from the onset. For example, for mothers with low self-efficacy, providing mothers with alternate activities to help them succeed at limiting television viewing, particularly during challenging situations or child behaviours, may be effective at increasing self-efficacy [10]. For mothers with high self-efficacy, positive reinforcement and feedback may help to maintain their self-efficacy. As interventions to increase parenting self-efficacy in other domains have been successful at improving parenting behaviours and child outcomes [26], developing health promotion programs that focus on enhancing or maintaining maternal self-efficacy for limiting children’s television viewing in this early childhood period are critical.

Mothers who rated their child’s temperament as ‘average’ or ‘more difficult or much more difficult than average’ were less likely to have persistently high self-efficacy for limiting children’s television viewing compared to those who rated their child’s temperament as ‘much easier than average’. The influence of difficult child temperament has previously been investigated in studies around parental feeding practices [27], picky eating [28], weight gain [29] and television viewing [19]. Specific to television viewing, findings by Thompson and colleagues identified that children perceived by their mother to have a more active or fussy temperament were exposed to more television than children with a less active temperament [19]. Drawing from qualitative work [30], it can be hypothesized that in these situations, parents may have opted to use the television to distract the child or calm him or her down or may have been less inclined to restrict television if the child wanted to watch. Supporting this hypothesis is recent findings from Radesky and colleagues [31]. In that study, infants with poor self-regulation (also considered a challenging child behaviour for mothers) at 9-months had higher television viewing time at 2 years-old than their peers with no/mild self-regulation problems.

However, it is also possible that children who exhibit challenging child behaviours (e.g. negative temperament, poor self-regulation) may reduce maternal self-efficacy by impacting on the quality of the mother-child relationship [32]. This negative mother-child relationship may in turn, result in mothers’ greater use of television over engagement in interactions with her child. Although there may be many mechanisms to explain the association between child temperament and maternal behaviours, our study highlights that it may be even more critical for mothers of infants with a difficult temperament to have support available (e.g. parenting skills and strategies) from early in their child’s life to help them enact alternative strategies to television use to distract, entertain or manage their child.

Although the greatest percentage of mothers decreased tertile ranking in self-efficacy for limiting children’s television viewing over time, it is important to note that approximately a quarter of mothers increased their self-efficacy ranking. This may have occurred because mothers were successful at limiting their child’s television viewing during the 15-month period and therefore experienced an increase in self-efficacy. Additionally, mothers who met the physical activity recommendations at T1 were more likely to demonstrate increased self-efficacy. It is possible that more active mothers were more able to provide opportunities for active play or may engage in more physical activity with their children rather than television viewing. It would be beneficial to further investigate mothers who show positive change in self-efficacy over time to identify how and why these changes occur. Such information would be invaluable for the development of future intervention strategies aiming to reduce television viewing in very young children.

This was the first study to assess the tracking of mothers’ self-efficacy for limiting her child’s television viewing in a relatively large sample; however it was not without limitations. For example, although the time between the two measures of self-efficacy spanned a period of rapid child development (4 months old – 19 months old), it is not known whether the pattern of movement would remain consistent over time. In other words, the direction of change in self-efficacy experienced over this relatively short period of time may not be reflective of what parents will experience as their child enters the preschool and primary school years. Additionally, the measures used within this study were all based on maternal self-report and consisted of only a few items as they were part of a larger study. In particular, children’s television viewing time was assessed with only one item. More comprehensive assessments and/or the use of objective measures (where possible) may provide less biased estimates. Finally, although this study aimed to capture hypothesized predictors of change in self-efficacy based on previous literature, it is possible that other broader variables not assessed in this study (e.g. maternal depression) may also significantly impact on maternal self-efficacy and children’s television viewing time.

It should be noted in the present study that more than half the mothers were highly educated, and many met the physical activity recommendations and engaged in quite low weekly television viewing at baseline compared to the Australian population [23, 33]. Thus, these findings may not translate to the general population. Investigating the tracking of maternal self-efficacy in more diverse populations, particularly those with lower levels of education, is desired. Finally, by dividing the data into tertiles (although a common approach for tracking studies [24, 25]) true stability may have over- or under-estimated if there was substantial movement around or within the cut-off points.

## Conclusion

Findings from the current study suggest support is needed to both increase and maintain maternal self-efficacy for limiting their children’s television viewing in the early childhood period, in order to reduce children’s later television viewing time. This may be particularly relevant for mothers of children with a difficult temperament. Promoting maternal engagement in physical activity may be a useful strategy for increasing maternal self-efficacy for limiting television viewing. Future research is needed to examine the long-term tracking of maternal self-efficacy and to identify strategies employed by mothers who possess and maintain high self-efficacy for limiting their child’s television viewing.

## Abbreviations

MPA: Moderate-intensity physical activity
MVPA: Moderate- to vigorous intensity physical activity
T1: Time point 1
T2: Time point 2
VPA: Vigorous-intensity physical activity
